# Combined effects of land use and hunting on distributions of tropical mammals

**DOI:** 10.1111/cobi.13459

**Published:** 2020-03-23

**Authors:** Juan Gallego‐Zamorano, Ana Benítez‐López, Luca Santini, Jelle P. Hilbers, Mark A. J. Huijbregts, Aafke M. Schipper

**Affiliations:** ^1^ Department of Environmental Science, Institute for Wetland and Water Research, Faculty of Science Radboud University P.O. Box 9010 Nijmegen NL‐6500 GL The Netherlands; ^2^ Integrative Ecology Group Estación Biológica de Doñana (EBD‐CSIC) Av. Americo Vespucio S/N Sevilla 41092 Spain; ^3^ PBL Netherlands Environmental Assessment Agency P.O. Box 30314 NL‐2500 GH The Hague The Netherlands; ^4^ National Research Council Institute of Research on Terrestrial Ecosystems (CNR‐IRET) Via Salaria km 29.300 Rome 00015 Italy

**Keywords:** biodiversity, conservation, defaunation, deforestation, overexploitation, tropics, biodiversidad, conservación, defaunación, deforestación, sobreexplotación, trópicos, 生物多样性, 保护, 动物丧失, 森林砍伐, 过度利用, 热带

## Abstract

Land use and hunting are 2 major pressures on biodiversity in the tropics. Yet, their combined impacts have not been systematically quantified at a large scale. We estimated the effects of both pressures on the distributions of 1884 tropical mammal species by integrating species’ range maps, detailed land‐use maps (1992 and 2015), species‐specific habitat preference data, and a hunting pressure model. We further identified areas where the combined impacts were greatest (hotspots) and least (coolspots) to determine priority areas for mitigation or prevention of the pressures. Land use was the main driver of reduced distribution of all mammal species considered. Yet, hunting pressure caused additional reductions in large‐bodied species’ distributions. Together, land use and hunting reduced distributions of species by 41% (SD 30) on average (year 2015). Overlap between impacts was only 2% on average. Land use contributed more to the loss of distribution (39% on average) than hunting (4% on average). However, hunting reduced the distribution of large mammals by 29% on average; hence, large mammals lost a disproportional amount of area due to the combination of both pressures. Gran Chaco, the Atlantic Forest, and Thailand had high levels of impact across the species (hotspots of area loss). In contrast, the Amazon and Congo Basins, the Guianas, and Borneo had relatively low levels of impact (coolspots of area loss). Overall, hunting pressure and human land use increased from 1992 to 2015 and corresponding losses in distribution increased from 38% to 41% on average across the species. To effectively protect tropical mammals, conservation policies should address both pressures simultaneously because their effects are highly complementary. Our spatially detailed and species‐specific results may support future national and global conservation agendas, including the design of post‐2020 protected area targets and strategies.

## Abstract 2

*Article impact statement*: Tropical mammals have lost on average 40% of their original distribution due to the combined effect of hunting pressure and human land use.

## Abstract (es)

Efectos Combinados del Uso de Suelo y la Caza en la Distribución de los Mamíferos Tropicales

**Resumen**

El uso de suelo y la caza son dos de las principales presiones ejercidas sobre la biodiversidad de los trópicos. Aun así, los impactos combinados que generan no han sido cuantificados sistemáticamente a gran escala. Estimamos los efectos de ambas presiones sobre la distribución de 1,884 especies de mamíferos tropicales al integrar mapas de distribución de las especies, mapas detallados del uso de suelo (de 1992 y 2015), datos de preferencia de hábitat específicos por especie y un modelo de presión de caza. Identificamos además las áreas en donde los impactos combinados eran mayores (puntos calientes) y menores (puntos fríos) para determinar las áreas prioritarias para la mitigación o prevención de dichas presiones. El uso de suelo fue el principal conductor de la reducción de la distribución para todas las especies de mamíferos que consideramos. Sin embargo, la presión por caza causó reducciones adicionales en la distribución de especies de gran tamaño. Juntas, el uso de suelo y la caza redujeron la distribución de las especies en un 41% (DS 30) en promedio (año 2015). El solapamiento entre los impactos fue, en promedio, sólo del 2%. El uso de suelo contribuyó más a la pérdida de la distribución (39%, en promedio) que la caza (4%, en promedio). A pesar de esto, en promedio la caza redujo la distribución de los mamíferos de gran tamaño en un 29%; por lo tanto, los grandes mamíferos perdieron una cantidad desproporcionada de área debido a la combinación de ambas presiones. El Gran Chaco, el Bosque Atlántico y Tailandia tuvieron niveles altos de impacto en todas las especies (puntos calientes de pérdida de área). Como contraste, las cuencas del Amazonas y el Congo, las Guayanas y Borneo tuvieron niveles relativamente bajos de impacto (puntos fríos de pérdida de área). En general, las presiones por caza y uso de suelo incrementaron desde 1992 a 2015 y las correspondientes pérdidas de distribución incrementaron de un 38% a un 41% en promedio para todas las especies. Para proteger de forma efectiva a los mamíferos tropicales, las políticas de conservación deberían considerar a ambas presiones de manera simultánea, pues sus efectos son altamente complementarios. Nuestros resultados espacialmente detallados y específicos para cada especie pueden respaldar las futuras agendas de conservación nacionales e internacionales, incluyendo el diseño de las estrategias y los objetivos de las áreas protegidas para después de 2020.

## 摘要

土地利用和狩猎是热带地区生物多样性面临的两大压力。然而, 它们的共同影响尚未在大尺度上得到系统的定量研究。我们通过整合物种分布范围、详细的土地利用地图 (1992 年及 2015 年) 、物种特异的栖息地偏好数据和狩猎压力模型, 估计了这两种压力对 1884 种热带哺乳动物分布的影响。我们进一步确定了二者共同影响最强 (热点) 和最弱 (冷点) 的地区, 以确定减缓或预防压力的优先区域。土地利用是所有哺乳动物分布减少的主要原因, 而狩猎压力则导致大型动物的分布范围进一步缩小。土地利用和狩猎使物种分布范围平均减少了 41% (标准差 30) (2015 年) 。而两种压力的影响平均只有 2% 的重叠范围。土地利用对物种分布减少的作用 (平均 39%) 大于狩猎 (平均 4%) 。然而, 狩猎使大型哺乳动物的分布平均减少了 29%; 因此, 大型哺乳动物在这两种压力的共同作用下分布区不成比例地发生退缩。格兰查科、大西洋森林和泰国为分布区缩小的热点地区, 对所有哺乳动物都有很大的影响。而亚马逊和刚果盆地、圭亚那及婆罗洲是影响相对较弱的地区。总的来说, 1992 年至 2015 年来, 狩猎压力和人类土地利用压力都越来越严重, 所有物种平均的分布区丧失也从 38% 增加到 41% 。为了有效地保护热带哺乳动物, 保护政策需同时应对这两种压力, 因为它们的作用是高度互补的。我们富有空间细节和物种特异性的研究结果可以支持未来的国家及全球保护议程, 包括 2020 年后保护地目标和战略的设计。**【翻译: 胡怡思; 审校: 聂永刚】**

## Introduction

Overexploitation and habitat loss due to agricultural activities are major pressures on biodiversity in the tropics (Maxwell et al. 2016). Recent estimates indicate mammal populations have been reduced by more than 80% and by 30% due to hunting pressure and land‐use change, respectively (Almeida‐Rocha et al. 2017; Benítez‐López et al. 2017). So far, most research has focused on quantifying the impacts of these 2 pressures separately (Almeida‐Rocha et al. 2017; Benítez‐López et al. 2017, 2019; de Lima et al. 2018), yet both threats typically act simultaneously. For example, deforestation and associated infrastructural development can improve hunters’ access to previously remote intact areas (Fa & Brown 2009; Abernethy et al. 2013; Laurance et al. 2017). Global conservation targets and actions also typically address one of the 2 pressures (e.g., Aichi Targets 4 and 5 [CBD 2010]) and may thus fall short in addressing overall conservation goals. Hence, studies addressing the combined impacts of land use and hunting are urgently needed.

Only a few researchers have quantified the combined effect of both pressures on tropical mammals (Brodie et al. 2015; Romero‐Muñoz et al. 2019). These authors found that the relative and combined effects of the 2 pressures differ among species and geographic areas, highlighting the relevance of looking at both pressures simultaneously to design effective conservation actions. However, previous studies were limited to a single region or based on a few species, and the combined effects of both pressures have not yet been comprehensively assessed across multiple mammal species at a large spatial extent. This information is urgently required for informing large‐scale conservation planning and prioritization by identifying disproportionally affected areas as well as pristine places where species are still relatively safe (hotspots vs. coolspots [e.g., Allan et al. 2019]).

We quantified the combined impact of land use and hunting on the geographic distributions of 1884 tropical mammal species. While land use may result in reductions in distribution due to habitat loss, hunting can lead to extirpations in areas that are otherwise suitable (Wilkie et al. 2011; Benítez‐López et al. 2017). Both pressures thus lead to a reduction in the distribution of wildlife species, which may compromise their persistence (Brook et al. 2008; Allan et al. 2019). We mapped habitat loss due to land use by combining species’ geographic range maps with land‐use maps and species‐specific habitat‐preference data. We quantified reductions of the distribution of each species due to hunting pressure as a function of distance to hunters’ access points, human population density, and body size of the species, which are major determinants of hunting impacts (Benítez‐López et al. 2017, 2019). Finally, we quantified reductions in the distribution due to both pressures combined and evaluated possible changes in the impacts of these pressures over the past decades (1992 to 2015).

## Methods

### Species Selection and Initial Distribution

**Figure 1:**
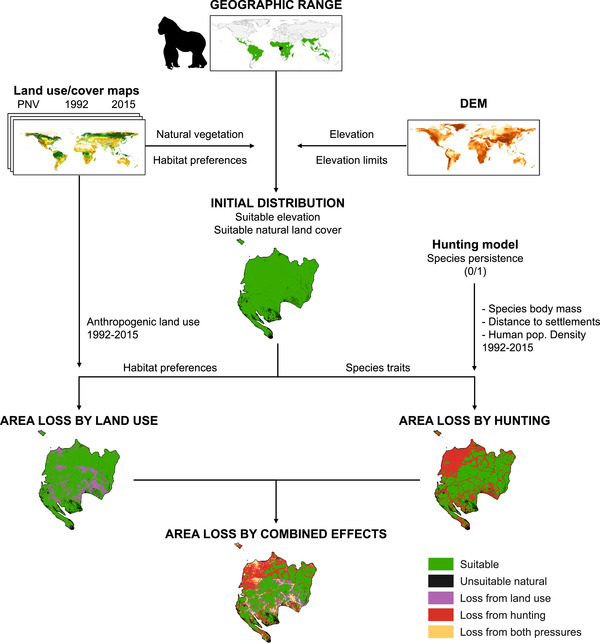
Steps in the model of distribution loss for an example species (Western gorilla [*Gorilla gorilla*]) (DEM, digital elevation model; suitable, areas suitable for the species; unsuitable natural, natural land cover not suitable for the species). Final map is total area of distribution loss of the species.

We selected mammal species with at least 95% of their geographic ranges in the tropics. We retrieved maps of the geographical ranges of all terrestrial mammal species from the IUCN (2017) and clipped these to the tropics based on the recently updated biomes map by Dinerstein et al. (2017). We considered 4 tropical biomes: Tropical and Subtropical Moist Broadleaf Forests, Tropical and Subtropical Dry Broadleaf Forests, Tropical and Subtropical Coniferous Forests, and Tropical and Subtropical Grasslands, Savannas, and Shrublands. This selection yielded 1884 species. Because geographic range maps are rather coarse representations of the distributions of species, we refined the range maps based on the species’ elevation limits and habitat (Brooks et al. 2019) (Fig. 1). For elevation we used the MERIT Digital Elevation Model (Yamazaki et al. 2017) at 10 arc seconds resolution (∼300 m at the equator) and selected areas within the elevation limits of the species as defined by the International Union for Conservation of Nature (IUCN 2017). For species lacking information on elevation limits, we assumed they occur across the entire elevational gradient within their range. We then filtered out unsuitable natural areas based on species’ habitat preferences and a natural land‐cover map. We compiled the natural land‐cover map (10 arc seconds resolution) by combining a land‐use map for 1992 (see below) with a map of potential natural vegetation (PNV) (Hengl et al. 2018). We used the cells with natural land cover from our 1992 land‐use map and assigned the vegetation type from the PNV map to the remaining (i.e., anthropogenic land uses) cells. We preferred this combined map for natural land cover over using only the PNV map because of the higher spatial resolution of the land‐use map (10 arc seconds as opposed to 30 arc seconds) and its more refined classification of natural land‐cover types. We then removed cells with unsuitable natural land cover from the species’ ranges based on species‐specific information on habitat preferences as provided by the IUCN Habitat Classification Scheme (level 2) and on a cross‐walk between the IUCN habitat classes and the natural land‐cover classes (Supporting Information) (Santini et al. 2019). The area remaining in the occurrence range of a species after the elevation and land‐cover filtering constituted our initial distribution estimate (i.e., baseline distribution) (Fig. 1).

### Area Loss Due to Land Use

For each species, we quantified area loss relative to their initial distribution due to land use based on land‐use maps for the years 1992 and 2015 (Fig. 1 & Supporting Information). We compiled the land‐use maps with the land‐use allocation routine from the GLOBIO 4 model (Schipper et al. 2019), in which we combined country‐level total areas of forestry and pasture with the recently released European Space Agency (ESA) climate change initiative (CCI) land‐cover maps (ESA 2017). These maps represent a consistent series of yearly land‐cover maps from 1992 to 2015 at a 10 arc seconds resolution. Cropland and urban areas are included in these maps, but pastures and forestry areas are not because they cannot be distinguished from natural grassland and forests (yet can be unsuitable for many species [Barona et al. 2010]). We, therefore, retrieved country‐level total areas of pasture and forestry representative for 1992 and 2015 from the Food and Agriculture Organisation (2016) and downscaled these onto the ESA maps for these same years with the GLOBIO 4 land‐use allocation routine (Schipper et al. 2019) (details in Supporting Information). Within each species’ distribution, we then quantified the total area of the anthropogenic land‐use types (i.e., urban areas, croplands, pastures, and forestry) unsuitable for the species based on species‐specific habitat preferences (Fig. 1 & Supporting Information) in the absence of any other pressure such as hunting. We considered the area loss due to land use as the sum of the cells lost due to all 4 land‐use types together.

### Area Loss by Hunting Pressure

To account for hunting pressure, we estimated the areas within each species’ initial distribution where it would likely be extirpated due to hunting. We used a mixed‐effects model with a binomial error distribution to quantify the species‐specific probability of persistence under hunting pressure as a function of various key determinants of hunting pressure, namely, the distance to hunters’ access points (settlements), human population density, and the species’ body mass. We fitted the model based on a data base with 3281 mammal abundance estimates of 296 species (from 51 families and 14 orders) from 163 studies and 114 papers that systematically compared abundance between hunted and unhunted sites within the tropics (Benítez‐López et al. 2017, 2019). Estimates were only included in this database if confounding factors were (virtually) absent or the same in the hunted area and the unhunted control site (Benítez‐López et al. 2017). This database is the most extensive database of the impact of hunting on species abundance in the tropics, in terms of location coverage (37 countries) and number of species (see above), and it covers the majority of families and the body mass range of our selection of tropical mammals (Benítez‐López et al. 2017, 2019) (Supporting Information). To estimate loss of distribution due to hunting, we transformed abundance data into occurrence (abundance > 0, *n* = 2873) and extirpation (abundance = 0, *n* = 408). We retrieved the distance to access points from the hunting database (Benítez‐López et al. 2017, 2019), human population density (matched as closely as possible to the year of the study) from CIESIN (2016), and body mass from the EltonTraits database (Wilman et al. 2014). We log_10_ transformed the continuous predictor variables before model fitting and included quadratic terms to account for potential nonlinear relationships. We specified as random effects country, study (typically encompassing the data from one article, but some articles report on multiple studies), and species to account for between‐country variation in hunting laws and policies, culture, taboos, and traditions (Ngoufo & Waltert 2014; Bobo et al. 2015) and to control for nonindependence in the data from the same study or species (Benítez‐López et al. 2019). Finally, we selected the most parsimonious model based on the Akaike information criterion (Supporting Information). The best model included distance to settlements and its quadratic term, human population density, and species’ body mass.

We then used the best model to predict for each tropical species the probability of persistence under hunting pressure within its distribution at a 30 arc seconds (∼1 km) resolution. Our predictions were based on the taxonomic identity of the species (captured by the random effect intercept species) and its body mass (species’ vulnerability to hunting pressure) combined with the distribution of context‐dependent drivers of hunting pressure in the species’ initial distribution (i.e., country, captured by the random effect intercept country; distance to settlements; and human population density). We retrieved data on human population density specific to 1992 (average between 1990 and 1995) and 2015 from CIESIN (CIESIN & CIAT 2005; CIESIN 2016) and a raster map of distance to the nearest settlement from Benitez‐Lopez et al. (2019). This represented a static view of the location of human settlements. To estimate the impacts of hunting on the distribution of the species, we transformed the probabilities of occurrence of each species as predicted by the hunting model into a binary variable (1, species potentially present; 0, species extirpated). These values were based on a probability threshold that maximized the true skills statistic (TSS) (Allouche et al. 2006), which assesses the predictive power of the model based on the sensitivity and specificity values (TSS = sensitivity + specificity − 1). The TSS range is from −1 (all predictions are wrong) to 1 (all predictions are correct). The transformation to a binary variable resulted in species‐specific 30 arc seconds maps of potential area loss due to hunting pressure in the initial species’ distribution (Fig. 1). Finally, we resampled the hunting‐impact maps (30 arc seconds) to the same spatial resolution as the land‐use impact maps (10 arc seconds).

### Combined Impacts of Both Pressures

To quantify the total reduction in the species’ distributions, we overlaid the maps of both pressures and identified the area lost due to hunting only, land use only, and the overlap of the 2 pressures (Fig. 1). We then calculated the combined impact of both pressures relative to the initial distribution for each species as

(1) $$P_{\mathrm{loss},i}=\left(\frac{A_{\mathrm{loss},i,\mathrm{LU}}+A_{\mathrm{loss},i,H}-A_{\mathrm{loss},i,\mathrm{LU}\cap H\;}}{A_{i}}\right)\;\times 100,$$

where *P*_loss,_ *_i_* is the area loss due to both pressures combined relative to the initial distribution (percentage) of species *i*, A_loss,_ *_i_*_,LU_ is the area loss (square kilometers) due to land use only, *A*_loss,_ *_i,_*_H_ is the loss (square kilometers) due to hunting only, *A*_loss_ *_,i_*_,LU∩H_ is the overlap in loss between the 2 pressures (square kilometers), and *A_i_* is the initial distribution (square kilometers).

We grouped our area‐loss results by species group based on body size: very small (<0.1 kg, *n* = 979 species), small (0.1–1 kg, *n =* 532), medium (1–10 kg, *n* = 291), and large (>10 kg, *n* = 82). We further calculated the average area loss across mammals from different continents (the Americas, Africa, and Asia). Finally, we compared the area losses from 1992 to 2015 to identify possible changes in the magnitude and relative importance of the 2 pressures over time.

### Hotspots and Coolspots of Area Loss

We defined hotspots and coolspots of area loss as areas with great (>90%) or small (<10%) distribution loss due to the combined pressures across the species per 0.25° (∼25 km) grid cell (for computation and visualization purposes). In each 0.25° cell, we divided the cumulative area lost by the cumulative initial area across all the species present in that cell:

(2) $$\;P_{\mathrm{loss},y}=\;\frac{\sum_{i=1}^{n}\;A_{\mathrm{loss},i,y}}{\sum_{i=1}^{n}\;A_{i,y}},$$

where *P*_loss,_ *_y_* is the average area loss (percentage) in cell *y* (0.25° resolution, ∼25 km^2^), *A*_loss_ *_,i,y_* is the area loss of species *i* in cell *y* (square kilometers), and *A_i,y_* is the initial area of species *i* in cell *y* (square kilometers). All the calculations were done using a Mollweide equal‐area projection in R 3.5.3 (R Development Core Team 2016). The code and data used for the analysis can be accessed at https://doi.org/10.17026/dans-zah-vs6x.

## Results

### Area Loss Due to Land Use and Hunting Pressure

**Figure 2:**
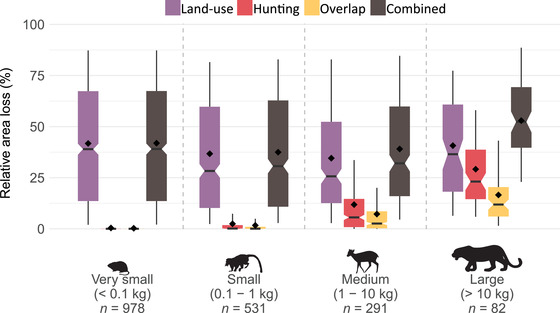
For 2015 distribution losses of 1884 species due to land use and hunting pressure (combined, sum of both pressures minus their overlap; diamonds, mean values per group; lower and upper box boundaries, 25th and 75th percentiles, respectively; thick horizontal line, median; notch, 95% CI around the estimate of the median; whiskers, 10–90 percentiles). Summary statistics are in Supporting Information.

On average across the species, distributions of the species declined 41% (SD 30) due to the combined impacts of hunting and land use (2% overlap between the 2 pressures [Supporting Information]). Land use resulted in an average loss of 39% of the initial distribution (SD 30) and hunting in a loss of 4% (SD 11). The smallest mammal species (<0.1 kg) were mostly affected by land use (loss of 42% [SD 31]) (Fig. 2), whereas area losses estimated for large species (>10 kg) were due to both land use (40% [SD 26]) and hunting pressure (29% [SD 21]). Hunting was the main pressure for 30% of the large species (Supporting Information). As a result, large mammals were the most affected group overall, showing an average area loss of 53% (SD 24) (Fig. 2) due to both pressures combined.

### Geographical Patterns of Area Loss

**Figure 3:**
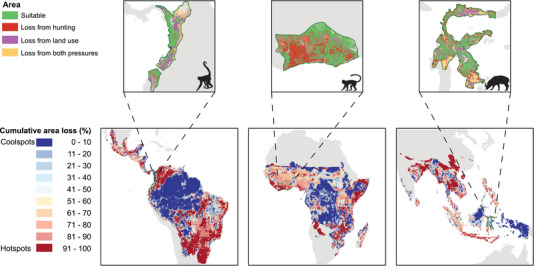
Upper panels, loss of distribution due to hunting, land use, and both for the brown‐headed spider monkey (*Ateles fusciceps*), Lowe's monkey (*Cercopithecus lowei*), and Sulawesi babirusa (Babyrousa celebensis) (from left to right) (suitable, areas suitable for the species). Lower panels, cumulative distribution loss of 1884 tropical mammal species due to land use and hunting relative to the cumulative area of their initial distribution.

Areas of great distribution loss (hotspots) across the species were identified in the Gran Chaco, Atlantic Forest, El Cerrado, northwestern part of South America, East Africa, Madagascar, Thailand, and Java. Areas with small loss (coolspots) were in the Amazon Basin, the Guianas, the Congo Basin, central Borneo, and Papua New Guinea (Fig. 3).

**Figure 4:**
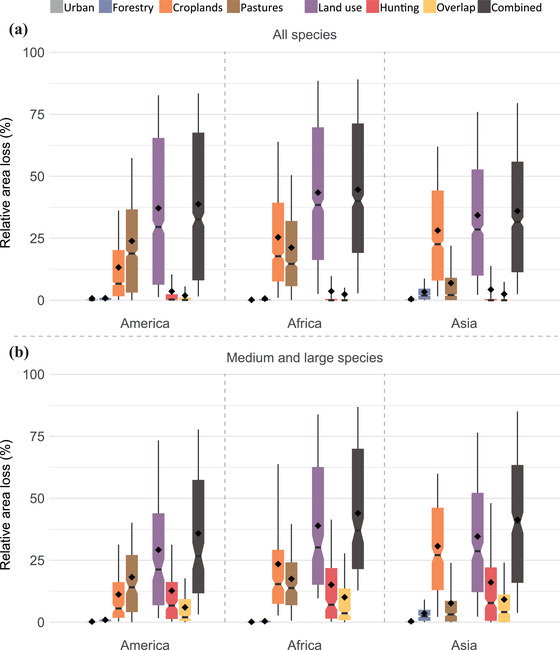
Losses in distribution size for (a) all species and (b) only medium (1–10 kg) and large mammals (>10 kg) due to land use and hunting pressure across the species by continent in 2015 (combined, sum of losses due to land use and hunting minus the overlapping areas; diamonds, mean values per group; lower and upper box boundaries, 25th and 75th percentiles; thick horizontal line, median; notch, 95% CI around estimate of the median; whiskers, 10–90 percentile). Summary statistics are in Supporting Information.

Estimated area losses were the greatest in Africa (average loss 46% [SD 30]), followed by the Americas (40% [SD 31]), and Asia (37% [SD 28]) (Fig. 4 & Supporting Information). Land use was the main driver of area loss on all continents, resulting in average losses ranging from 35% in Asia to 45% in Africa (Fig. 4a). These losses were mainly driven by croplands in Africa and Asia (26% [SD 24] and 28% [SD 23] loss) and by pastures in the Americas (24% [SD 22] loss). Mammal species were predicted to be extirpated by hunting across 5% (SD 13) of their initial distribution in Asia (Fig. 4a) and 3–4% on the other continents (Supporting Information). When looking only at medium and large species, up to 16% (SD 20) of the initial distribution was under high hunting pressure in Asia and 15% (SD 18) and 13% (SD 17) in Africa and the Americas, respectively (Fig. 4b & Supporting Information).

### Changes Over Time

**Figure 5:**
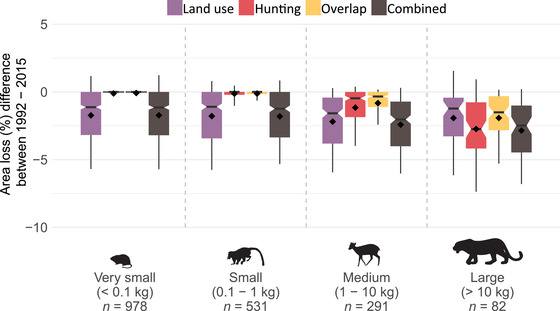
Changes in distribution‐size losses due to land use and hunting pressure from 1992 to 2015 (negative values, loss of area from 1992 to 2015; positive values, gain of area; diamonds, mean values per group; lower and upper box boundaries, 25th and 75th percentiles, thick horizontal line, median; notch, 95% CI around the estimate of the median; whiskers, 10–90 percentile). Summary statistics in Supporting Information.

Losses in distribution increased from a mean of 38% (SD 31) in 1992 to 41% (SD 30) in 2015 (Supporting Information). Some species increased their distribution (i.e., 423 species), for example, in Ethiopia where the area of pasture decreased from 448,000 km^2^ in 1992 to 288,000 km^2^ in 2015 (FAO 2016). Yet, most species experienced further loss (i.e., 1387 species), mainly driven by land‐use change (Fig. 5 & Supporting Information). For medium‐sized species, hunting pressure also increased over time, leading to additional reductions in distribution. Large species also experienced increases in the impacts of both pressures; increases were larger for hunting than for land‐use impacts (Fig. 5).

## Discussion

To our knowledge, we are the first to quantify the combined impact of land use and hunting pressure on the distributions of mammals across the entire tropical region. Our results suggest that tropical mammals lost on average 40% of their distribution due to these 2 pressures combined (Figs. 2, 4, and 5), whereby land use is responsible for the largest share (39%) (Figs. 2, 4, & Supporting Information). This is in agreement with a recent analysis of threats to biodiversity based on IUCN threat status information, showing that more species are threatened by crop and livestock farming than by hunting (Maxwell et al. 2016). However, our results also indicate hunting is a major pressure on large mammals (Supporting Information), extirpating populations across ∼30% of their distribution on average (Fig. 2), confirming that hunting renders large species locally extinct (Ripple et al. 2016, 2019).

For the largest species, the increase in hunting impacts was larger than the increase in land‐use impacts (Fig. 5). We further found that the impacts of both pressures are highly species specific (Figs. 2, 3, & Supporting Information). For example, Lowe's monkey (*Cercopithecus lowei*), a generalist species, was primarily affected by hunting (but see Linder & Oates [2011]), whereas the brown‐headed spider monkey (*Ateles fusciceps*), a forest specialist, was affected most by deforestation and land‐use change. We found a relatively small overlap between the impacts of the 2 pressures (Figs. 2, 4, 5, & Supporting Information), reflecting that hunting mainly takes place in remaining areas of natural habitat that are not yet affected by land use (Ripple et al. 2016, 2019; Benítez‐López et al. 2017, 2019). Hence, both pressures are largely complementary in yielding losses in the distribution of the species. As a result, large mammals in particular lost a disproportional amount of area due to both pressures combined (Fig. 2 & Supporting Information). Overall, we considered our area loss estimates conservative (optimistic) because we did not account for additional effects of land use, such as fragmentation or edge effects, that may cause small area remnants to be functionally lost (Pe'er et al. 2014). Additionally, we did not consider access points other than settlements (e.g., roads), and we accounted only for hunting impacts that cause extirpations, whereas many hunted mammal populations could be largely reduced without necessarily being extirpated (Benítez‐López et al. 2017, 2019). These reduced populations may become functionally extinct (i.e., nonviable or no longer contributing to ecosystem functioning) before being totally extirpated from an area. Consequently, the effect of hunting may be larger than estimated here, in line with the finding that our model predicted larger hunting impacts with a higher threshold when the predicted probabilities of occurrence were transformed to a binary variable (i.e., using a threshold corresponding with minimizing the error of predicting local extinctions) (Supporting Information).

Our results further showed clear spatial variation in the effects of the 2 pressures. At the level of continents, we found that pasture may remove 24% of natural habitat in the Americas, whereas only 7% may be removed in Asia (Fig. 4a & Supporting Information), reflecting that extensive grazing is one of the major drivers of deforestation in South America (Barona et al. 2010). Furthermore, hunting impacts were bigger in Asia and Africa than in the Americas (Fig. 4b & Supporting Information), where bushmeat hunting is largely driven by demand for medicinal products, ornamentals, or trophy products (Ripple et al. 2016) and species are accessible and have higher population densities. In some areas with high distribution loss (hotspots), there were very few tropical species (e.g., southern Africa [Angola] and central China) (Fig. 3 & Supporting Information). Yet, hotspots of loss also occurred in species‐rich areas, such as some parts of South America, East Africa, and Southeast Asia (Fig. 3 & Supporting Information). Our results are in line with previous research demonstrating that tropical mammals in the Gran Chaco, the Atlantic Forest, and Java are threatened by both land‐use change and hunting (Symes et al. 2018; Romero‐Muñoz et al. 2019).

In contrast, the Amazon and Congo Basins, the Guianas, Borneo, and Papua New Guinea had relatively small loss of distribution across the species (coolspots), which is in line with results of previous efforts to map human impacts on biodiversity (Venter et al. 2016; Allan et al. 2019; Benítez‐López et al. 2019; Schipper et al. 2019). With the ongoing increase of human activities in tropical areas, remaining intact places may be compromised in the future (Watson et al. 2018; Allan et al. 2019). Indeed, our results show that both land use and hunting pressure increased over the past decades (Fig. 5 & Supporting Information). Therefore, we suggest conservation efforts focus on reducing or mitigating these pressures in hotspots and preventing further degradation in coolspots, which is an urgent priority for current global efforts to halt the ongoing biodiversity crisis (Watson et al. 2018). Limiting the construction of new roads and enforcing laws against illegal deforestation, hunting, and wildlife trade may contribute to this goal (Peres 2005; Ripple et al. 2016).

In 2020 the Convention on Biological Diversity will adopt a post‐2020 global biodiversity framework, which calls for evidence‐based conservation targets and strategies (CBD 2019). Our results demonstrate the importance of accounting for the combined effect of land use and hunting on medium and large species because their effects are highly complementary (i.e., the 2 pressures affect different parts of the species’ distribution and their relative importance differs among species) (Figs. 2, 3, 4, and 5) (Brodie et al. 2015; Symes et al. 2018). The magnitude of the impacts combined with the poor level of protection of remaining wilderness areas (Di Marco et al. 2019) points to the need to increase the level of protection if tropical mammals are to be conserved (Peres 2005; Geldmann et al. 2019). Protected areas need to be strengthened, for example, through law enforcement, effective prosecution, and community engagement (Geldmann et al. 2019), to ensure their effectiveness in halting both pressures simultaneously and protect tropical mammals more effectively.

## Supporting information

Description of land‐use allocation by the GLOBIO model (Appendix S1) and of crosswalk between the GLOBIO land‐use map and the IUCN habitat classification (Appendix S2), comparison between hunting database and selected tropical mammal species (Appendix S3), area loss by land use and hunting with 2 thresholds (Appendix S4), area loss due to different pressures (Appendix S5), patterns of tropical mammal species richness (Appendix S6), species affected by land use or hunting pressure as main driver of distribution reduction (Appendix S7), model selection results for the binomial hunting model (Appendix S8), mean area loss due to different pressures (Appendix S9), and cross‐walk from IUCN habitat classes to ESA CII and GLOBIO classes 11 (Appendix S10) are available online. The authors are solely responsible for the content and functionality of these materials. Queries (other than absence of the material) should be directed to the corresponding author.
