# Supplementary material for: Combined effects of land use and hunting on distributions of tropical mammals
Source: Conserv Biol. 2020 Mar 23;34(5):1271–80. doi: 10.1111/cobi.13459 (PMC7540261; doi:10.1111/cobi.13459)
Supplement: Supplementary file 1 — Description of land‐use allocation by the GLOBIO model (Appendix S1) and of crosswalk between the GLOBIO land‐use map and the IUCN habitat classification (Appendix S2), comparison between hunting database and selected tropical mammal species (Appendix S3), area loss by land use and hunting with 2 thresholds (Appendix S4), area loss due to different pressures (Appendix S5), patterns of tropical mammal species richness (Appendix S6), species affected by land use or hunting pressure as main driver of distribution reduction (Appendix S7), model selection results for the binomial hunting model (Appendix S8), mean area loss due to different pressures (Appendix S9), and cross‐walk from IUCN habitat classes to ESA CII and GLOBIO classes 11 (Appendix S10) are available online. The authors are solely responsible for the content and functionality of these materials. Queries (other than absence of the material) should be directed to the corresponding author. [file COBI-34-1271-s001.pdf]

SUPPLEMENTARY MATERIAL

Hunting pressure complements land use in reducing the distributions of tropical mammals

Contents

Appendix S1. Description of the land use allocation by the GLOBIO model 2

Appendix S2. Description of the crosswalk between the GLOBIO land-use map and the IUCN habitat classification 3

Figure S1. Comparison between the hunting database and the selected tropical mammal species.. 4

Figure S2. Area loss by land use and hunting with two different thresholds for the hunting model based on a) maximum specificity and b) maximum sensitivity........... 5

Figure S3. Area loss by different pressures for 1992 and 2015 6

Figure S4. Patterns of tropical mammal species richness 7

Figure S5. Percentage of species affected by land use or hunting pressure as main driver of distribution size reduction 8

Table S1. Model selection results for the binomial hunting model............................. 9

Table S2. Mean values and standard deviation of area loss by different pressures ... 10

Table S3. Cross-walk from IUCN habitat classes to ESA CII and GLOBIO classes 11

Appendix S1. Description of the land-use allocation by the GLOBIO model

The GLOBIO model (version 4) includes a 10 arc-seconds (~300m resolution at the equator) land-use allocation procedure to capture the spatial heterogeneity of land use (Kim et al. 2018). Three types of inputs are required: 1) regional totals or demands ("claims") of each land-use type, expressed in terms of area (km^2); 2) a base map with the natural land cover, and 3) map layers quantifying the suitability of each grid cell for each land-use type. In this study, we distinguished five anthropogenic land-use types that match the IUCN habitat classification scheme, i.e., arable land, pastureland, plantations, urban areas and secondary vegetation (tropical heavily degraded former forest). For arable lands and urban areas, we directly used the ESA's CCI land cover maps for the years 1992 and 2015. Because the ESA's land cover map does not include data on pastures and plantations, we used data from the FAO to obtain the claims for those land use types. The FAO provides total areas (km^2) of different land use practices per country and year. For pasture and rangeland we used area of *Permanent and Temporary meadows and pastures*, and for plantations the area of *Planted forest*, based on country-specific data reported for 1992 and 2015 (FAO 2016). We then used the GLOBIO 4 allocation routine to allocate the claims of pasture and forestry (in which forestry takes precedence over pasture), using the corresponding suitability layers (Kim et al. 2018) and using the ESA CCI maps of the respective years as a base maps. Thus, in essence we superimposed pasture and forestry land onto the ESA CCI land-cover maps of 1992 and 2015. Further details on the GLOBIO land-use allocation routine can be found in Schipper et al. (2019).

Appendix S2. Description of the crosswalk between the GLOBIO land-use map and the IUCN habitat classification

We created this crosswalk in a two-step procedure. We first made an initial connection between the GLOBIO land-use classes and the level 1 and 2 classes of the Habitat Classification Scheme of the IUCN (IUCN 2015), based on similarities in definitions (i.e. vegetation types and climate). Second, to further refine the initial cross-walk, we matched the definitions of the IUCN habitats with the biomes from Dinerstein et al. (2017) and identified which land-use classes felt inside the different biomes. In addition, to account for habitats occurring at high altitudes (e.g. Forest-Subtropical/Tropical Moist Montane (IUCN habitat 1.9), occurs generally above c.1200m), we used a high-resolution digital elevation map to identify the land uses occurring over the threshold of 1200 m that the IUCN defines.

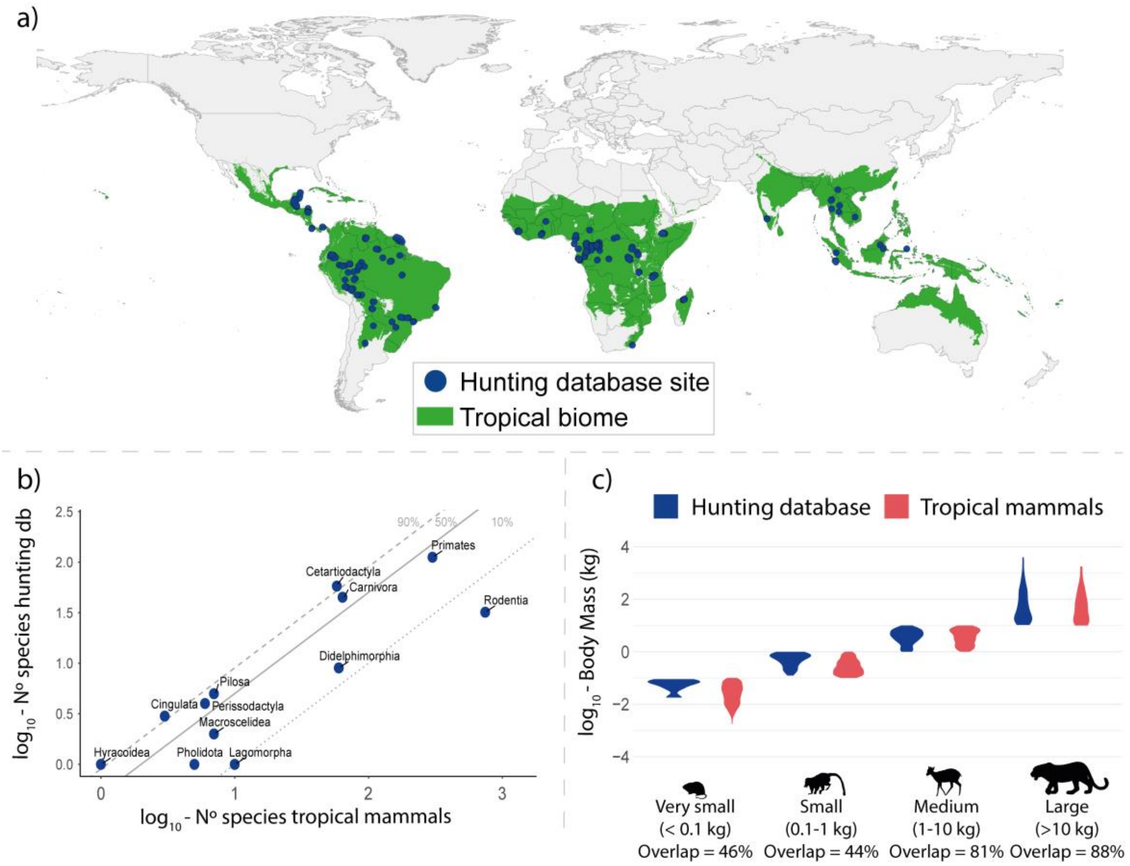

Figure S1 Comparison between the hunting database and the selection of tropical mammals in terms of a) location, b) number of species and c) body mass coverage. a) Location of study sites in the hunting database and the coverage of the tropical biome that we used to subset the selection of tropical mammals. b) Relationship between the number of species represented in our database (N = 296) and the number of tropical species for which we extrapolated our models (N = 1,884) for 12 orders. Lines show proportions of 10%, 50% and 90% (dotted, solid, dashed, respectively) representation. c) Violin plot showing the representation and percentage overlap between the hunting database and the selection of tropical mammals by different body size groups.

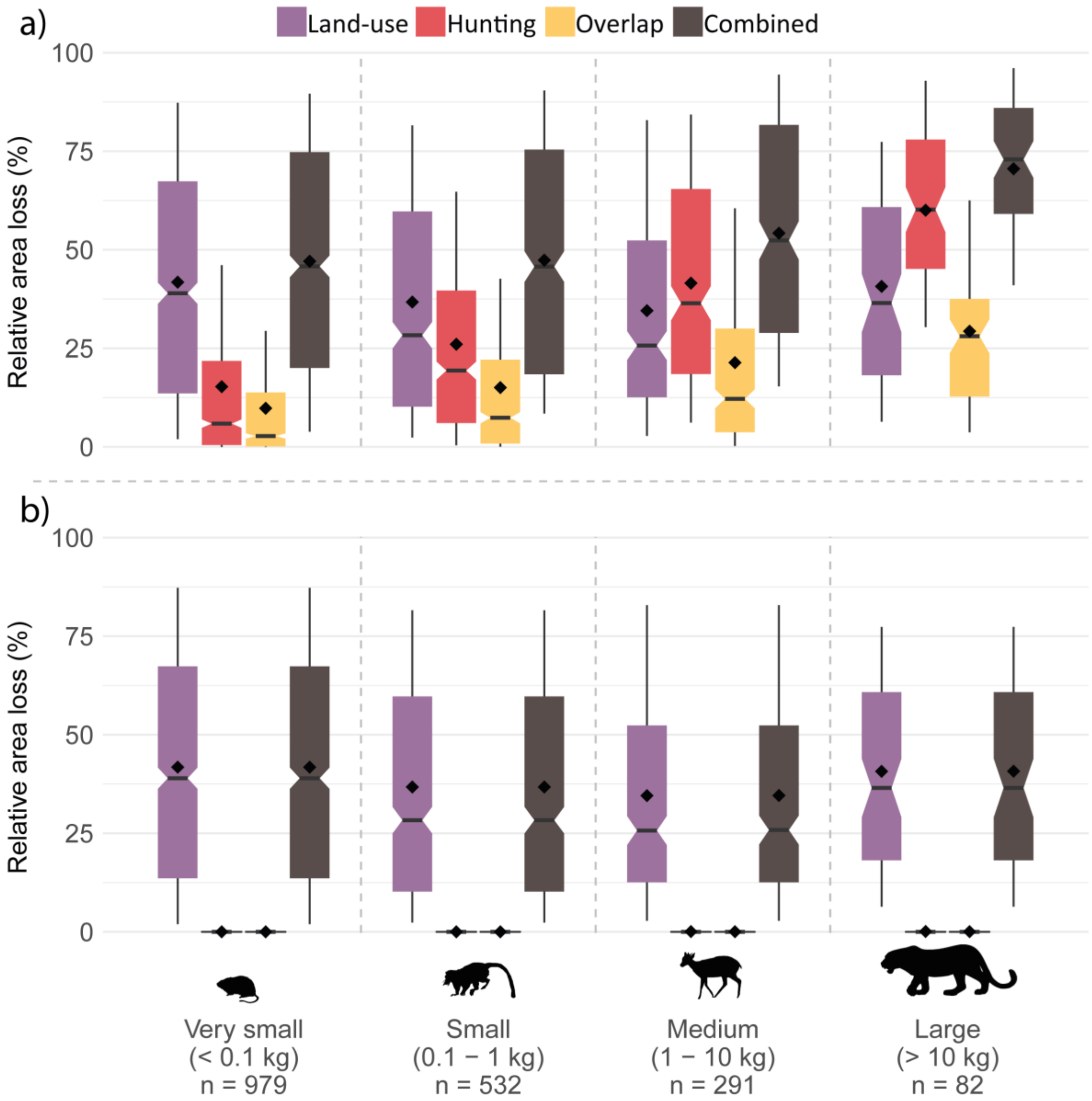

Figure S2 Reductions in distribution (%) by land use and hunting with two different thresholds for the hunting model based on a) maximum specificity (minimizing the error of predicting local extinction) and b) maximum sensitivity (minimizing the error of predicting occurrence). The model performance of these scenarios was measured by the TSS which ranges between -1 (all predictions are wrong) and 1 (all predictions are correct). In both scenarios the TSS was lower than obtained with maximizing both sensitivity and specificity (as presented in the main text), i.e., we obtained TSS of 0.55 for a) and 0.10 for b), compared to a TSS of 0.72 for the default model.

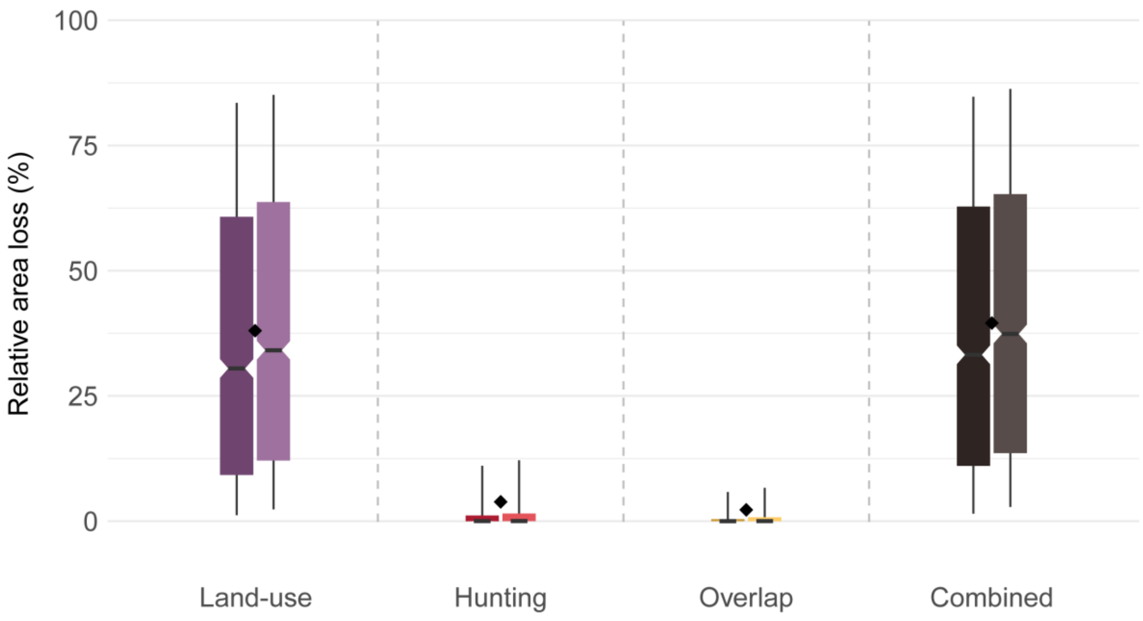

Figure S3 Losses in species' distributions (%) due to land use and hunting pressure for 1992 (left-hand boxes) and 2015 (right-hand boxes). The combined effect is the result of the sum of the losses due to land use and hunting minus the overlapping areas. The black diamonds represent the mean values per group; lower and upper box boundaries are 25th and 75th percentiles, the black thick line inside the box is the median, the notch represents the 95% confidence interval around the median and the whiskers the 10-90% percentile.

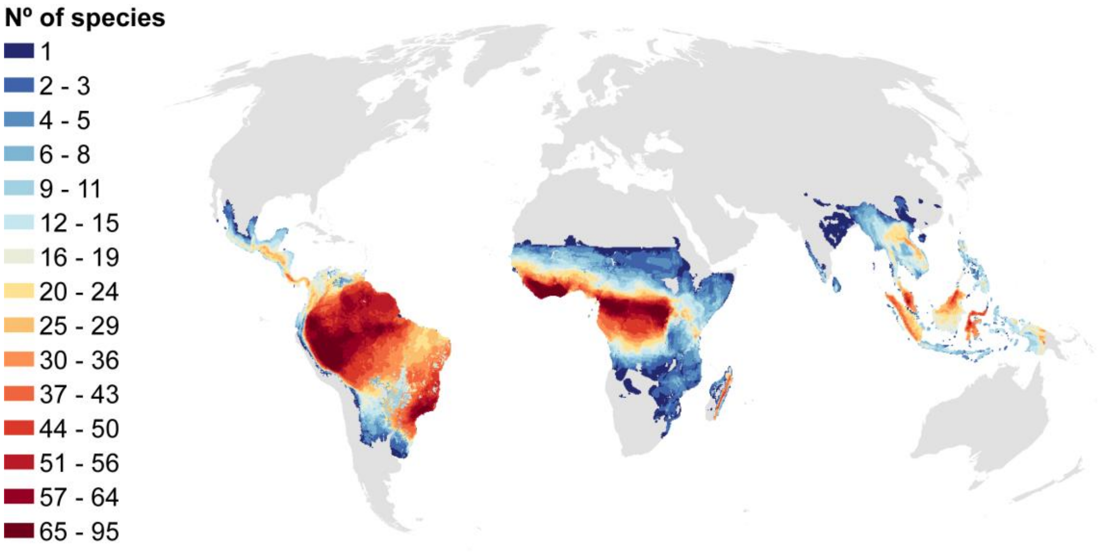

Figure S4 Number of species per 0.25º grid cell (~25 km) in the tropical biomes included in our analysis.

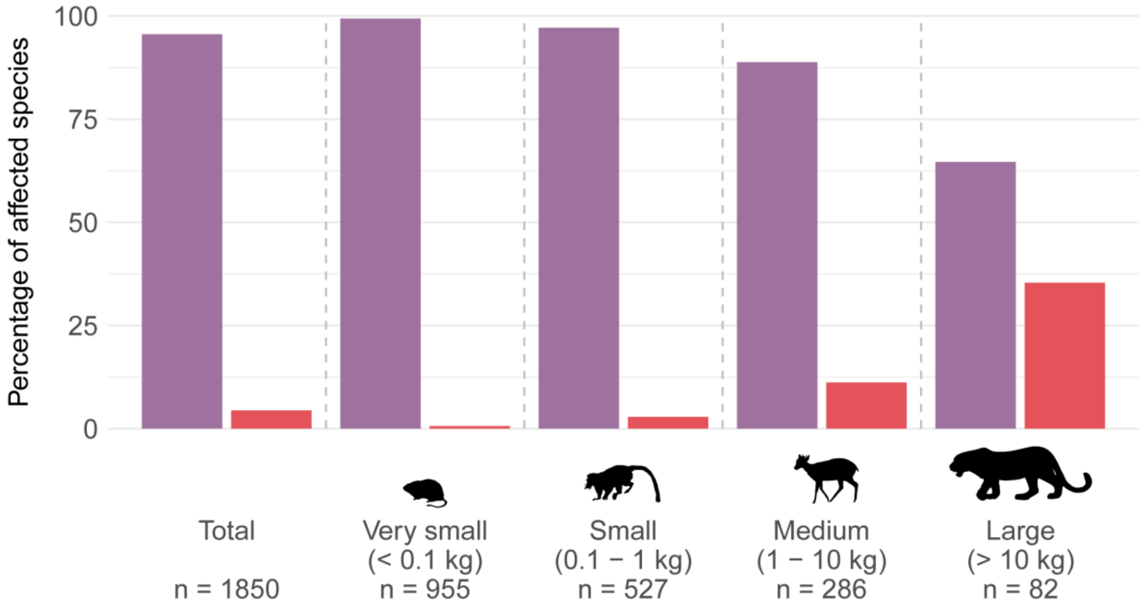

Figure S5 Relative number of species (%) per body mass group affected by land use (purple bar) or hunting pressure (red bar) as main driver of losses in distribution for the year 2015. Per species the main driver was calculated as the pressure that removed the most area relative to its extent of suitable area. Species that were not affected by either pressure or equally affected were discarded. The percentage is calculated based on the number of species per body mass group.

Table S1. Model selection results for the binomial hunting model. AIC was used to select the best model, which was applied to calculate the hunting pressure per species. BM: body mass, Dist: distance to hunters' access points, PopDens: human population density. Random effects were species identity, country of the study and study identity.

Variables	Df	logLik	AIC	ΔAIC
**BM + Dist + Dist2 + PopDens**	**8**	**-977.53**	**1971.06**	**0**
BM + Dist + Dist2 + PopDens + PopDens2	9	-977.52	1973.04	1.98
BM + Dist + Dist2	7	-980.9	1975.81	4.75
Dist + Dist2 + PopDens	7	-987.69	1989.38	18.32
Dist + Dist2 + PopDens + PopDens2	8	-987.68	1991.36	20.31
Dist + Dist2	6	-991.44	1994.88	23.83
BM + Dist + PopDens + PopDens2	8	-993.14	2002.28	31.22
BM + Dist	6	-999.64	2011.28	40.22
Dist + PopDens + PopDens2	7	-1003.2	2020.4	49.34
Dist	5	-1009.91	2029.82	58.77
BM + PopDens	6	-1086.59	2185.19	214.13
BM + PopDens + PopDens2	7	-1086.94	2187.89	216.83
PopDens	5	-1095.5	2201.01	229.95
PopDens + PopDens2	6	-1095.4	2202.8	231.75
BM	5	-1099.01	2208.02	236.96
Null model (only random effects)	4	-1107.24	2222.48	251.42

Table S2. Mean values and standard deviation of area loss by different pressures. Mean values and standard deviation of area loss by different pressures for different body size groups, continents, and years. Size- and continent-specific values are for the year 2015.

grouping	size	N	Combined	Overlap	Hunting	Land use	Croplands	Pastures	Forestry	Urban
Size	large	82	52.95 ± 24.34	16.56 ± 16.47	29.16 ± 21.31	40.70 ± 26.44	25.83 ± 21.77	16.42 ± 17.51	2.46 ± 4.02	0.32 ± 0.93
	medium	291	39.07 ± 28.90	7.20 ± 12.46	11.84 ± 16.77	34.55 ± 28.63	23.92 ± 21.76	12.75 ± 15.20	2.47 ± 3.91	0.41 ± 0.88
	small	532	37.42 ± 30.24	1.68 ± 5.18	2.46 ± 6.75	36.67 ± 30.11	20.59 ± 20.49	17.36 ± 21.43	1.99 ± 2.96	0.90 ± 4.82
	very small	979	41.86 ± 30.83	0.27 ± 1.68	0.41 ± 2.09	41.73 ± 30.85	23.33 ± 22.68	18.56 ± 21.49	2.03 ± 4.56	0.59 ± 2.41
Continent	Africa	570	46.19 ± 30.57	2.79 ± 8.93	4.02 ± 11.40	44.97 ± 30.77	26.30 ± 23.57	21.73 ± 21.39	0.84 ± 3.50	0.24 ± 0.65
	America	647	39.50 ± 31.32	1.93 ± 5.24	3.45 ± 8.81	38.03 ± 31.35	13.74 ± 15.34	24.05 ± 22.66	0.97 ± 1.48	0.98 ± 4.58
	Asia	667	37.04 ± 28.28	2.65 ± 8.54	4.52 ± 12.62	35.24 ± 27.93	28.48 ± 23.11	6.78 ± 11.52	4.30 ± 5.12	0.65 ± 2.57
Total	2015	1884	40.65 ± 30.27	2.45 ± 7.71	4.00 ± 11.07	39.14 ± 30.25	22.76 ± 21.93	17.23 ± 20.55	2.11 ± 4.05	0.64 ± 3.12
	1992	1884	38.35 ± 30.52	2.05 ± 6.64	3.59 ± 10.27	36.82 ± 30.47	21.58 ± 21.82	16.88 ± 20.60	1.25 ± 2.99	0.34 ± 1.91

Table S3. Cross-walk from IUCN habitat classes to ESA CII and GLOBIO classes

IUCN Code	IUCN Description	ESA Code	ESA Description	GLOBIO Code	GLOBIO Description	Natural/ Anthropogenic
**1.1**	Forest – Boreal	60	Tree cover, broadleaved, deciduous, closed to open (>15%)	60	Tree cover, broadleaved, deciduous, closed to open (>15%)	Natural
**1.1**	Forest – Boreal	61	Tree cover, broadleaved, deciduous, closed (>40%)	61	Tree cover, broadleaved, deciduous, closed (>40%)	Natural
**1.1**	Forest – Boreal	62	Tree cover, broadleaved, deciduous, open (15-40%)	62	Tree cover, broadleaved, deciduous, open (15-40%)	Natural
**1.1**	Forest – Boreal	70	Tree cover, needleleaved, evergreen, closed to open (>15%)	70	Tree cover, needle-leaved, evergreen, closed to open (>15%)	Natural
**1.1**	Forest – Boreal	71	Tree cover, needleleaved, evergreen, closed (>40%)	71	Tree cover, needle-leaved, evergreen, closed (>40%)	Natural
**1.1**	Forest – Boreal	72	Tree cover, needleleaved, evergreen, open (15-40%)	72	Tree cover, needle-leaved, evergreen, open (15-40%)	Natural
**1.1**	Forest – Boreal	80	Tree cover, needleleaved, deciduous, closed to open (>15%)	80	Tree cover, needle-leaved, deciduous, closed to open (>15%)	Natural
**1.1**	Forest – Boreal	81	Tree cover, needleleaved, deciduous, closed (>40%)	81	Tree cover, needle-leaved, deciduous, closed (>40%)	Natural
**1.1**	Forest – Boreal	82	Tree cover, needleleaved, deciduous, open (15-40%)	82	Tree cover, needle-leaved, deciduous, open (15-40%)	Natural
**1.1**	Forest – Boreal	90	Tree cover, mixed leaf type (broadleaved and needleleaved)	90	Tree cover, mixed leaf type (broadleaved and needle-leaved)	Natural
**1.2**	Forest – Subarctic	60	Tree cover, broadleaved, deciduous, closed to open (>15%)	60	Tree cover, broadleaved, deciduous, closed to open (>15%)	Natural
**1.2**	Forest – Subarctic	62	Tree cover, broadleaved, deciduous, open (15-40%)	62	Tree cover, broadleaved, deciduous, open (15-40%)	Natural
**1.2**	Forest – Subarctic	70	Tree cover, needleleaved, evergreen, closed to open (>15%)	70	Tree cover, needle-leaved, evergreen, closed to open (>15%)	Natural
**1.2**	Forest – Subarctic	72	Tree cover, needleleaved, evergreen, open (15-40%)	72	Tree cover, needle-leaved, evergreen, open (15-40%)	Natural
**1.2**	Forest – Subarctic	80	Tree cover, needleleaved, deciduous, closed to open (>15%)	80	Tree cover, needle-leaved, deciduous, closed to open (>15%)	Natural

IUCN Code	IUCN Description	ESA Code	ESA Description	GLOBIO Code	GLOBIO Description	Natural/ Anthropogenic
**1.2**	Forest - Subarctic	82	Tree cover, needleleaved, deciduous, open (15-40%)	82	Tree cover, needle-leaved, deciduous, open (15-40%)	Natural
**1.3**	Forest – Subantarctic	60	Tree cover, broadleaved, deciduous, closed to open (>15%)	60	Tree cover, broadleaved, deciduous, closed to open (>15%)	Natural
**1.3**	Forest – Subantarctic	62	Tree cover, broadleaved, deciduous, open (15-40%)	62	Tree cover, broadleaved, deciduous, open (15-40%)	Natural
**1.3**	Forest – Subantarctic	70	Tree cover, needleleaved, evergreen, closed to open (>15%)	70	Tree cover, needle-leaved, evergreen, closed to open (>15%)	Natural
**1.3**	Forest – Subantarctic	72	Tree cover, needleleaved, evergreen, open (15-40%)	72	Tree cover, needle-leaved, evergreen, open (15-40%)	Natural
**1.3**	Forest – Subantarctic	80	Tree cover, needleleaved, deciduous, closed to open (>15%)	80	Tree cover, needle-leaved, deciduous, closed to open (>15%)	Natural
**1.3**	Forest – Subantarctic	82	Tree cover, needleleaved, deciduous, open (15-40%)	82	Tree cover, needle-leaved, deciduous, open (15-40%)	Natural
**1.4**	Forest – Temperate	50	Tree cover, broadleaved, evergreen, closed to open (>15%)	50	Tree cover, broadleaved, evergreen, closed to open (>15%)	Natural
**1.4**	Forest – Temperate	60	Tree cover, broadleaved, deciduous, closed to open (>15%)	60	Tree cover, broadleaved, deciduous, closed to open (>15%)	Natural
**1.4**	Forest – Temperate	61	Tree cover, broadleaved, deciduous, closed (>40%)	61	Tree cover, broadleaved, deciduous, closed (>40%)	Natural
**1.4**	Forest – Temperate	62	Tree cover, broadleaved, deciduous, open (15-40%)	62	Tree cover, broadleaved, deciduous, open (15-40%)	Natural
**1.4**	Forest – Temperate	70	Tree cover, needleleaved, evergreen, closed to open (>15%)	70	Tree cover, needle-leaved, evergreen, closed to open (>15%)	Natural
**1.4**	Forest – Temperate	71	Tree cover, needleleaved, evergreen, closed (>40%)	71	Tree cover, needle-leaved, evergreen, closed (>40%)	Natural
**1.4**	Forest – Temperate	72	Tree cover, needleleaved, evergreen, open (15-40%)	72	Tree cover, needle-leaved, evergreen, open (15-40%)	Natural
**1.4**	Forest – Temperate	80	Tree cover, needleleaved, deciduous, closed to open (>15%)	80	Tree cover, needle-leaved, deciduous, closed to open (>15%)	Natural
**1.4**	Forest – Temperate	81	Tree cover, needleleaved, deciduous, closed (>40%)	81	Tree cover, needle-leaved, deciduous, closed (>40%)	Natural
**1.4**	Forest – Temperate	82	Tree cover, needleleaved, deciduous, open (15-40%)	82	Tree cover, needle-leaved, deciduous, open (15-40%)	Natural

IUCN Code	IUCN Description	ESA Code	ESA Description	GLOBIO Code	GLOBIO Description	Natural/ Anthropogenic
**1.4**	Forest – Temperate	90	Tree cover, mixed leaf type (broadleaved and needleleaved)	90	Tree cover, mixed leaf type (broadleaved and needle-leaved)	Natural
**1.4**	Forest – Temperate	100	Mosaic tree and shrub (>50%) / herbaceous cover (<50%)	100	Mosaic tree and shrub (>50%) / herbaceous cover (<50%)	Natural
**1.5**	Forest - Subtropical/Tropical Dry	50	Tree cover, broadleaved, evergreen, closed to open (>15%)	50	Tree cover, broadleaved, evergreen, closed to open (>15%)	Natural
**1.5**	Forest - Subtropical/Tropical Dry	60	Tree cover, broadleaved, deciduous, closed to open (>15%)	60	Tree cover, broadleaved, deciduous, closed to open (>15%)	Natural
**1.5**	Forest - Subtropical/Tropical Dry	61	Tree cover, broadleaved, deciduous, closed (>40%)	61	Tree cover, broadleaved, deciduous, closed (>40%)	Natural
**1.5**	Forest - Subtropical/Tropical Dry	62	Tree cover, broadleaved, deciduous, open (15-40%)	62	Tree cover, broadleaved, deciduous, open (15-40%)	Natural
**1.5**	Forest - Subtropical/Tropical Dry	70	Tree cover, needleleaved, evergreen, closed to open (>15%)	70	Tree cover, needle-leaved, evergreen, closed to open (>15%)	Natural
**1.5**	Forest - Subtropical/Tropical Dry	72	Tree cover, needleleaved, evergreen, open (15-40%)	72	Tree cover, needle-leaved, evergreen, open (15-40%)	Natural
**1.5**	Forest - Subtropical/Tropical Dry	80	Tree cover, needleleaved, deciduous, closed to open (>15%)	80	Tree cover, needle-leaved, deciduous, closed to open (>15%)	Natural
**1.5**	Forest - Subtropical/Tropical Dry	90	Tree cover, mixed leaf type (broadleaved and needleleaved)	90	Tree cover, mixed leaf type (broadleaved and needle-leaved)	Natural
**1.6**	Forest - Subtropical/Tropical Moist Lowland	50	Tree cover, broadleaved, evergreen, closed to open (>15%)	50	Tree cover, broadleaved, evergreen, closed to open (>15%)	Natural
**1.6**	Forest - Subtropical/Tropical Moist Lowland	60	Tree cover, broadleaved, deciduous, closed to open (>15%)	60	Tree cover, broadleaved, deciduous, closed to open (>15%)	Natural
**1.6**	Forest - Subtropical/Tropical Moist Lowland	61	Tree cover, broadleaved, deciduous, closed (>40%)	61	Tree cover, broadleaved, deciduous, closed (>40%)	Natural
**1.6**	Forest - Subtropical/Tropical Moist Lowland	62	Tree cover, broadleaved, deciduous, open (15-40%)	62	Tree cover, broadleaved, deciduous, open (15-40%)	Natural
**1.6**	Forest - Subtropical/Tropical Moist Lowland	70	Tree cover, needleleaved, evergreen, closed to open (>15%)	70	Tree cover, needle-leaved, evergreen, closed to open (>15%)	Natural
**1.6**	Forest - Subtropical/Tropical Moist Lowland	80	Tree cover, needleleaved, deciduous, closed to open (>15%)	80	Tree cover, needle-leaved, deciduous, closed to open (>15%)	Natural
**1.6**	Forest - Subtropical/Tropical Moist Lowland	90	Tree cover, mixed leaf type (broadleaved and needleleaved)	90	Tree cover, mixed leaf type (broadleaved and needle-leaved)	Natural

IUCN Code	IUCN Description	ESA Code	ESA Description	GLOBIO Code	GLOBIO Description	Natural/ Anthropogenic
**1.7**	Forest - Subtropical/Tropical Mangrove Vegetation Above High Tide Level	160	Tree cover, flooded, saline water	160	Tree cover, flooded, fresh or brackish water	Natural
**1.7**	Forest - Subtropical/Tropical Mangrove Vegetation Above High Tide Level	170	Tree cover, flooded, saline water	170	Tree cover, flooded, saline water	Natural
**1.7**	Forest - Subtropical/Tropical Mangrove Vegetation Above High Tide Level	180	Shrub or herbaceous cover, flooded, fresh/saline/brakish water	180	Shrub or herbaceous cover, flooded, fresh/saline/brackish water	Natural
**1.8**	Forest - Subtropical/Tropical Swamp	160	Tree cover, flooded, fresh or brakish water	160	Tree cover, flooded, fresh or brackish water	Natural
**1.9**	Forest - Subtropical/Tropical Moist Montane	50	Tree cover, broadleaved, evergreen, closed to open (>15%)	50	Tree cover, broadleaved, evergreen, closed to open (>15%)	Natural
**1.9**	Forest - Subtropical/Tropical Moist Montane	60	Tree cover, broadleaved, deciduous, closed to open (>15%)	60	Tree cover, broadleaved, deciduous, closed to open (>15%)	Natural
**1.9**	Forest - Subtropical/Tropical Moist Montane	61	Tree cover, broadleaved, deciduous, closed (>40%)	61	Tree cover, broadleaved, deciduous, closed (>40%)	Natural
**1.9**	Forest - Subtropical/Tropical Moist Montane	62	Tree cover, broadleaved, deciduous, open (15-40%)	62	Tree cover, broadleaved, deciduous, open (15-40%)	Natural
**1.9**	Forest - Subtropical/Tropical Moist Montane	70	Tree cover, needleleaved, evergreen, closed to open (>15%)	70	Tree cover, needle-leaved, evergreen, closed to open (>15%)	Natural
**1.9**	Forest - Subtropical/Tropical Moist Montane	71	Tree cover, needleleaved, evergreen, closed (>40%)	71	Tree cover, needle-leaved, evergreen, closed (>40%)	Natural
**1.9**	Forest - Subtropical/Tropical Moist Montane	90	Tree cover, mixed leaf type (broadleaved and needleleaved)	90	Tree cover, mixed leaf type (broadleaved and needle-leaved)	Natural
**2.1**	Savanna – Dry	110	Mosaic herbaceous cover (>50%) / tree and shrub (<50%)	110	Mosaic herbaceous cover (>50%) / tree and shrub (<50%)	Natural
**2.1**	Savanna – Dry	120	Shrubland	120	Shrubland	Natural
**2.1**	Savanna – Dry	121	Evergreen shrubland	121	Evergreen shrubland	Natural
**2.1**	Savanna – Dry	122	Deciduous shrubland	122	Deciduous shrubland	Natural
**2.1**	Savanna – Dry	130	Grassland	130	Grassland	Natural
**2.2**	Savanna – Moist	180	Shrub or herbaceous cover, flooded, fresh/saline/brakish water	180	Shrub or herbaceous cover, flooded, fresh/saline/brackish water	Natural
**2.2**	Savanna – Moist	100	Mosaic tree and shrub (>50%) / herbaceous cover (<50%)	100	Mosaic tree and shrub (>50%) / herbaceous cover (<50%)	Natural

IUCN Code	IUCN Description	ESA Code	ESA Description	GLOBIO Code	GLOBIO Description	Natural/ Anthropogenic
**2.2**	Savanna – Moist	110	Mosaic herbaceous cover (>50%) / tree and shrub (<50%)	110	Mosaic herbaceous cover (>50%) / tree and shrub (<50%)	Natural
**3.1**	Shrubland – Subarctic	120	Shrubland	120	Shrubland	Natural
**3.1**	Shrubland – Subarctic	121	Evergreen shrubland	121	Evergreen shrubland	Natural
**3.1**	Shrubland – Subarctic	122	Deciduous shrubland	122	Deciduous shrubland	Natural
**3.2**	Shrubland – Subantarctic	120	Shrubland	120	Shrubland	Natural
**3.2**	Shrubland – Subantarctic	121	Evergreen shrubland	121	Evergreen shrubland	Natural
**3.2**	Shrubland – Subantarctic	122	Deciduous shrubland	122	Deciduous shrubland	Natural
**3.3**	Shrubland – Boreal	120	Shrubland	120	Shrubland	Natural
**3.3**	Shrubland – Boreal	121	Evergreen shrubland	121	Evergreen shrubland	Natural
**3.3**	Shrubland – Boreal	122	Deciduous shrubland	122	Deciduous shrubland	Natural
**3.4**	Shrubland – Temperate	100	Mosaic tree and shrub (>50%) / herbaceous cover (<50%)	100	Mosaic tree and shrub (>50%) / herbaceous cover (<50%)	Natural
**3.4**	Shrubland – Temperate	110	Mosaic herbaceous cover (>50%) / tree and shrub (<50%)	110	Mosaic herbaceous cover (>50%) / tree and shrub (<50%)	Natural
**3.4**	Shrubland – Temperate	120	Shrubland	120	Shrubland	Natural
**3.4**	Shrubland – Temperate	121	Evergreen shrubland	121	Evergreen shrubland	Natural
**3.4**	Shrubland – Temperate	122	Deciduous shrubland	122	Deciduous shrubland	Natural
**3.5**	Shrubland - Subtropical/Tropical Dry	100	Mosaic tree and shrub (>50%) / herbaceous cover (<50%)	100	Mosaic tree and shrub (>50%) / herbaceous cover (<50%)	Natural
**3.5**	Shrubland - Subtropical/Tropical Dry	110	Mosaic herbaceous cover (>50%) / tree and shrub (<50%)	110	Mosaic herbaceous cover (>50%) / tree and shrub (<50%)	Natural
**3.5**	Shrubland - Subtropical/Tropical Dry	120	Shrubland	120	Shrubland	Natural
**3.5**	Shrubland - Subtropical/Tropical Dry	121	Evergreen shrubland	121	Evergreen shrubland	Natural
**3.5**	Shrubland - Subtropical/Tropical Dry	122	Deciduous shrubland	122	Deciduous shrubland	Natural
**3.6**	Shrubland - Subtropical/Tropical Moist	100	Mosaic tree and shrub (>50%) / herbaceous cover (<50%)	100	Mosaic tree and shrub (>50%) / herbaceous cover (<50%)	Natural
**3.6**	Shrubland - Subtropical/Tropical Moist	110	Mosaic herbaceous cover (>50%) / tree and shrub (<50%)	110	Mosaic herbaceous cover (>50%) / tree and shrub (<50%)	Natural
**3.6**	Shrubland - Subtropical/Tropical Moist	120	Shrubland	120	Shrubland	Natural

IUCN Code	IUCN Description	ESA Code	ESA Description	GLOBIO Code	GLOBIO Description	Natural/ Anthropogenic
**3.6**	Shrubland - Subtropical/Tropical Moist	121	Evergreen shrubland	121	Evergreen shrubland	Natural
**3.7**	Shrubland - Subtropical/Tropical High Altitude	100	Mosaic tree and shrub (>50%) / herbaceous cover (<50%)	100	Mosaic tree and shrub (>50%) / herbaceous cover (<50%)	Natural
**3.7**	Shrubland - Subtropical/Tropical High Altitude	110	Mosaic herbaceous cover (>50%) / tree and shrub (<50%)	110	Mosaic herbaceous cover (>50%) / tree and shrub (<50%)	Natural
**3.7**	Shrubland - Subtropical/Tropical High Altitude	120	Shrubland	120	Shrubland	Natural
**3.7**	Shrubland - Subtropical/Tropical High Altitude	121	Evergreen shrubland	121	Evergreen shrubland	Natural
**3.7**	Shrubland - Subtropical/Tropical High Altitude	122	Deciduous shrubland	122	Deciduous shrubland	Natural
**3.7**	Shrubland - Subtropical/Tropical High Altitude	150	Sparse vegetation (tree, shrub, herbaceous cover) (<15%)	150	Sparse vegetation (tree, shrub, herbaceous cover) (<15%)	Natural
**3.8**	Shrubland - Mediterranean-type Shrubby Vegetation	120	Shrubland	120	Shrubland	Natural
**3.8**	Shrubland - Mediterranean-type Shrubby Vegetation	121	Evergreen shrubland	121	Evergreen shrubland	Natural
**3.8**	Shrubland - Mediterranean-type Shrubby Vegetation	122	Deciduous shrubland	122	Deciduous shrubland	Natural
**4.1**	Grassland – Tundra	130	Grassland	130	Grassland	Natural
**4.1**	Grassland – Tundra	140	Lichens and mosses	140	Lichens and mosses	Natural
**4.1**	Grassland – Tundra	150	Sparse vegetation (tree, shrub, herbaceous cover) (<15%)	150	Sparse vegetation (tree, shrub, herbaceous cover) (<15%)	Natural
**4.1**	Grassland – Tundra	152	Sparse shrub (<15%)	152	Sparse shrub (<15%)	Natural
**4.1**	Grassland – Tundra	180	Shrub or herbaceous cover, flooded, fresh/saline/brakish water	180	Shrub or herbaceous cover, flooded, fresh/saline/brackish water	Natural
**4.2**	Grassland – Subarctic	130	Grassland	130	Grassland	Natural
**4.2**	Grassland – Subarctic	140	Lichens and mosses	140	Lichens and mosses	Natural
**4.2**	Grassland – Subarctic	150	Sparse vegetation (tree, shrub, herbaceous cover) (<15%)	150	Sparse vegetation (tree, shrub, herbaceous cover) (<15%)	Natural
**4.2**	Grassland – Subarctic	180	Shrub or herbaceous cover, flooded, fresh/saline/brakish water	180	Shrub or herbaceous cover, flooded, fresh/saline/brackish water	Natural

IUCN Code	IUCN Description	ESA Code	ESA Description	GLOBIO Code	GLOBIO Description	Natural/ Anthropogenic
**4.3**	Grassland – Subantarctic	130	Grassland	130	Grassland	Natural
**4.4**	Grassland – Temperate	130	Grassland	130	Grassland	Natural
**4.5**	Grassland - Subtropical/Tropical Dry	130	Grassland	130	Grassland	Natural
**4.6**	Grassland - Subtropical/Tropical Seasonally Wet/Flooded	180	Shrub or herbaceous cover, flooded, fresh/saline/brakish water	180	Shrub or herbaceous cover, flooded, fresh/saline/brackish water	Natural
**4.7**	Grassland - Subtropical/Tropical High Altitude	130	Grassland	130	Grassland	Natural
**5.1**	Wetlands (inland) - Permanent Rivers/Streams/Creeks (includes waterfalls)	210	Water bodies	210	Water bodies	Natural
**5.1**	Wetlands (inland) - Tundra Wetlands (incl. pools and temporary waters from snowmelt)	210	Water bodies	210	Water bodies	Natural
**5.11**	Wetlands (inland) - Alpine Wetlands (includes temporary waters from snowmelt)	210	Water bodies	210	Water bodies	Natural
**5.12**	Wetlands (inland) - Geothermal Wetlands	210	Water bodies	210	Water bodies	Natural
**5.13**	Wetlands (inland) - Permanent Inland Deltas	210	Water bodies	210	Water bodies	Natural
**5.14**	Wetlands (inland) - Permanent Saline, Brackish or Alkaline Lakes	210	Water bodies	210	Water bodies	Natural
**5.15**	Wetlands (inland) - Seasonal/Intermittent Saline, Brackish or Alkaline Lakes and Flats	210	Water bodies	210	Water bodies	Natural
**5.16**	Wetlands (inland) - Permanent Saline, Brackish or Alkaline Marshes/Pools	210	Water bodies	210	Water bodies	Natural
**5.17**	Wetlands (inland) - Seasonal/Intermittent Saline, Brackish or Alkaline Marshes/Pools	210	Water bodies	210	Water bodies	Natural
**5.18**	Wetlands (inland) - Karst and Other Subterranean Hydrological Systems (inland)	NA	NA	255	NA	Natural

IUCN Code	IUCN Description	ESA Code	ESA Description	GLOBIO Code	GLOBIO Description	Natural/ Anthropogenic
**5.2**	Wetlands (inland) - Seasonal/Intermittent/Irregular Rivers/Streams/Creeks	210	Water bodies	210	Water bodies	Natural
**5.3**	Wetlands (inland) - Shrub Dominated Wetlands	180	Shrub or herbaceous cover, flooded, fresh/saline/brakish water	180	Shrub or herbaceous cover, flooded, fresh/saline/brackish water	Natural
**5.4**	Wetlands (inland) - Bogs, Marshes, Swamps, Fens, Peatlands	180	Shrub or herbaceous cover, flooded, fresh/saline/brakish water	180	Shrub or herbaceous cover, flooded, fresh/saline/brackish water	Natural
**5.5**	Wetlands (inland) - Permanent Freshwater Lakes (over 8ha)	210	Water bodies	210	Water bodies	Natural
**5.6**	Wetlands (inland) - Seasonal/Intermittent Freshwater Lakes (over 8ha)	210	Water bodies	210	Water bodies	Natural
**5.7**	Wetlands (inland) - Permanent Freshwater Marshes/Pools (under 8ha)	210	Water bodies	210	Water bodies	Natural
**5.8**	Wetlands (inland) - Seasonal/Intermittent Freshwater Marshes/Pools (under 8ha)	210	Water bodies	210	Water bodies	Natural
**5.9**	Wetlands (inland) - Freshwater Springs and Oases	210	Water bodies	210	Water bodies	Natural
**6**	Rocky areas (eg. inland cliffs, mountain peaks)	200	Bare areas	200	Bare areas	Natural
**6**	Rocky areas (eg. inland cliffs, mountain peaks)	201	Consolidated bare areas	201	Consolidated bare areas	Natural
**8.1**	Desert – Hot	200	Bare areas	200	Bare areas	Natural
**8.1**	Desert – Hot	201	Consolidated bare areas	201	Consolidated bare areas	Natural
**8.1**	Desert – Hot	202	Unconsolidated bare areas	202	Unconsolidated bare areas	Natural
**8.1**	Desert – Hot	150	Sparse vegetation (tree, shrub, herbaceous cover) (<15%)	150	Sparse vegetation (tree, shrub, herbaceous cover) (<15%)	Natural
**8.1**	Desert – Hot	151	Sparse tree (<15%)	151	Sparse tree (<15%)	Natural
**8.1**	Desert – Hot	152	Sparse shrub (<15%)	152	Sparse shrub (<15%)	Natural
**8.1**	Desert – Hot	153	Sparse vegetation herbaceous cover (<15%)	153	Sparse herbaceous cover (<15%)	Natural
**8.2**	Desert – Temperate	200	Bare areas	200	Bare areas	Natural

IUCN Code	IUCN Description	ESA Code	ESA Description	GLOBIO Code	GLOBIO Description	Natural/ Anthropogenic
**8.2**	Desert – Temperate	201	Consolidated bare areas	201	Consolidated bare areas	Natural
**8.2**	Desert – Temperate	202	Unconsolidated bare areas	202	Unconsolidated bare areas	Natural
**8.2**	Desert – Temperate	150	Sparse vegetation (tree, shrub, herbaceous cover) (<15%)	150	Sparse vegetation (tree, shrub, herbaceous cover) (<15%)	Natural
**8.2**	Desert – Temperate	151	Sparse tree (<15%)	151	Sparse tree (<15%)	Natural
**8.2**	Desert – Temperate	152	Sparse shrub (<15%)	152	Sparse shrub (<15%)	Natural
**8.2**	Desert – Temperate	153	Sparse vegetation herbaceous cover (<15%)	153	Sparse herbaceous cover (<15%)	Natural
**8.3**	Desert – Cold	200	Bare areas	200	Bare areas	Natural
**8.3**	Desert – Cold	201	Consolidated bare areas	201	Consolidated bare areas	Natural
**8.3**	Desert – Cold	202	Unconsolidated bare areas	202	Unconsolidated bare areas	Natural
**8.3**	Desert – Cold	150	Sparse vegetation (tree, shrub, herbaceous cover) (<15%)	150	Sparse vegetation (tree, shrub, herbaceous cover) (<15%)	Natural
**8.3**	Desert – Cold	151	Sparse tree (<15%)	151	Sparse tree (<15%)	Natural
**8.3**	Desert – Cold	152	Sparse shrub (<15%)	152	Sparse shrub (<15%)	Natural
**8.3**	Desert – Cold	153	Sparse vegetation herbaceous cover (<15%)	153	Sparse herbaceous cover (<15%)	Natural
**8.3**	Desert – Cold	140	Lichens and mosses	140	Lichens and mosses	Natural
**14.1**	Artificial/Terrestrial - Arable Land	10	Cropland, rainfed	10	Cropland, rainfed	Anthropogenic
**14.1**	Artificial/Terrestrial - Arable Land	11	Cropland, rainfed, Herbaceous cover	11	Cropland, rainfed, Herbaceous cover	Anthropogenic
**14.1**	Artificial/Terrestrial - Arable Land	12	Cropland, rainfed, Tree or shrub cover	12	Cropland, rainfed, Tree or shrub cover	Anthropogenic
**14.1**	Artificial/Terrestrial - Arable Land	20	Cropland, irrigated or post-flooding	20	Cropland, irrigated or post-flooding	Anthropogenic
**14.1**	Artificial/Terrestrial - Arable Land	20	Cropland, irrigated or post-flooding	2	Cropland	Anthropogenic
**14.1**	Artificial/Terrestrial - Arable Land	20	Cropland, irrigated or post-flooding	231	Medium intensity cropland	Anthropogenic
**14.1**	Artificial/Terrestrial - Arable Land	20	Cropland, irrigated or post-flooding	232	High intensity cropland	Anthropogenic
**14.2**	Artificial/Terrestrial – Pastureland	130	Grassland	3	Pasture	Anthropogenic
**14.2**	Artificial/Terrestrial – Pastureland	130	Grassland	4	Rangeland	Anthropogenic
**14.3**	Artificial/Terrestrial – Plantations	12	Cropland, rainfed, Tree or shrub cover	5	Forestry	Anthropogenic

IUCN Code	IUCN Description	ESA Code	ESA Description	GLOBIO Code	GLOBIO Description	Natural/ Anthropogenic
**14.4**	Artificial/Terrestrial - Rural Gardens	30	Mosaic cropland (>50%) / natural vegetation (tree, shrub, herbaceous cover) (<50%)	30	Mosaic cropland (>50%) / natural vegetation (tree, shrub, herbaceous cover) (<50%)	Anthropogenic
**14.4**	Artificial/Terrestrial - Rural Gardens	40	Mosaic natural vegetation (tree, shrub, herbaceous cover) (>50%) / cropland (<50%)	40	Mosaic natural vegetation (tree, shrub, herbaceous cover) (>50%) / cropland (<50%)	Anthropogenic
**14.5**	Artificial/Terrestrial - Urban Areas	190	Urban areas	190	Urban areas	Anthropogenic
**14.6**	Artificial/Terrestrial - Subtropical/Tropical Heavily Degraded Former Forest	30	Mosaic cropland (>50%) / natural vegetation (tree, shrub, herbaceous cover) (<50%)	5	Forestry	Anthropogenic
**14.6**	Artificial/Terrestrial - Subtropical/Tropical Heavily Degraded Former Forest	30	Mosaic cropland (>50%) / natural vegetation (tree, shrub, herbaceous cover) (<50%)	6	Secondary vegetation	Anthropogenic

References supplementary

Dinerstein, E., Olson, D., Joshi, A., Vynne, C., Burgess, N.D., Wikramanayake, E., Hahn, N., Palminteri, S., Hedao, P., Noss, R., Hansen, M., Locke, H., Ellis, E.C., Jones, B., Barber, C.V., Hayes, R., Kormos, C., Martin, V., Crist, E., Sechrest, W., Price, L., Baillie, J.E.M., Weeden, D., Suckling, K., Davis, C., Sizer, N., Moore, R., Thau, D., Birch, T., Potapov, P., Turubanova, S., Tyukavina, A., de Souza, N., Pintea, L., Brito, J.C., Llewellyn, O.A., Miller, A.G., Patzelt, A., Ghazanfar, S.A., Timberlake, J., Klöser, H., Shennan-Farpón, Y., Kindt, R., Lillesø, J.-P.B., van Breugel, P., Graudal, L., Voge, M., Al-Shammari, K.F. & Saleem, M. (2017). An Ecoregion-Based Approach to Protecting Half the Terrestrial Realm. *Bioscience*, 67, 534–545. doi:10.1093/biosci/bix014

FAO. (2016). *FAOSTAT Land domain*. Food and Agriculture Organization of the Nations. URL:http://www.fao.org/faostat/en/#data/RL

IUCN. (2015). International union for conservation of nature. Habitat Classification Scheme Version, 3, 1.

Kim, H., Rosa, I.M.D., Alkemade, R., Leadley, P., Hurtt, G., Popp, A., van Vuuren, D.P., Anthoni, P., Arneth, A., Baisero, D., Caton, E., Chaplin-Kramer, R., Chini, L., De Palma, A., Di Fulvio, F., Di Marco, M., Espinoza, F., Ferrier, S., Fujimori, S., Gonzalez, R.E., Gueguen, M., Guerra, C., Harfoot, M., Harwood, T.D., Hasegawa, T., Haverd, V., Havlik, P., Hellweg, S., Hill, S.L.L., Hirata, A., Hoskins, A.J., Janse, J.H., Jetz, W., Johnson, J.A., Krause, A., Leclère, D., Martins, I.S., Matsui, T., Merow, C., Obersteiner, M., Ohashi, H., Poulter, B., Purvis, A., Quesada, B., Rondinini, C., Schipper, A.M., Sharp, R., Takahashi, K., Thuiller, W., Titeux, N., Visconti, P., Ware, C., Wolf, F. & Pereira, H.M. (2018).

A protocol for an intercomparison of biodiversity and ecosystem services models using harmonized land-use and climate scenarios. *Geosci. Model Dev.*, 11, 4537–4562. doi:10.5194/gmd-11-4537-2018

Schipper, A.M., Hilbers, J.P., Meijer, J.R., Antão, L.H., Benítez-López, A., de Jonge, M.M.J., Leemans, L.H., Scheper, E., Alkemade, R., Doelman, J.C., Mylius, S., Stehfest, E., van Vuuren, D.P., van Zeist, W.-J. & Huijbregts, M.A.J. (2019). Projecting terrestrial biodiversity intactness with GLOBIO 4. *Glob. Chang. Biol.*, 0. doi:10.1111/gcb.14848
